# Long non-coding RNA PVT1, a molecular sponge for miR-149, contributes aberrant metabolic dysfunction and inflammation in IL-1β-simulated osteoarthritic chondrocytes

**DOI:** 10.1042/BSR20180576

**Published:** 2018-09-28

**Authors:** Yangxue Zhao, Jiang Zhao, Xufeng Guo, Jiang She, Yongjun Liu

**Affiliations:** Department of Orthopaedics, the Ninth Hospital of Xi’an, Xi’an, Shaanxi 710054, P.R. China

**Keywords:** inflammation, lncRNA PVT1, metabolic dysfunction, miR-149, Osteoarthritis

## Abstract

Osteoarthritis (OA), a common form of degenerative joint disease, is typified by inflammatory response and the loss of cartilage matrix. Long non-coding RNAs (lncRNAs) are emerging as a new player in gene regulation and exert critical roles in diverse physiologic and pathogenic processes including OA. The lncRNA plasmacytoma variant translocation 1 (PVT1) has been implicated in cancer, diabetes and septic acute kidney injury. Recent research confirmed the elevation of PVT1 in patients with OA. However, its role in the development of OA remains poorly elucidated. In the present study, high expression of PVT1 was observed in cartilage of OA patients and IL-1β-stimulated chondrocytes. Moreover, cessation of PVT1 expression dramatically reversed the inhibition of IL-1β on collagen II and aggrecan expression, but suppressed IL-1β-induced elevation of matrix metalloproteinases (MMPs), including MMP-3, MMP-9 and MMP-13. Simultaneously, PVT1 inhibition also antagonized the production of inflammatory cytokines upon IL-1β stimulation, including prostaglandin E2 (PGE2), NO, IL-6, IL-8 and TNF-α. Further molecular mechanism analysis identified PVT1 as an endogenous sponge RNA that could directly bind to miR-149 and repress its expression and activity. More importantly, miR-149 inhibition reversed the protective roles of PVT1 cessation in attenuating IL-1β-evoked matrix aberrant catabolism and inflammation. Together, this research confirms that lowering PVT1 expression may ameliorate the progression of OA by alleviating cartilage imbalance toward catabolism and inflammatory response, thus supporting a promising therapeutic strategy against OA.

## Introduction

Osteoarthritis (OA) ranks as the most prevalent form of articulating joint diseases among elder, especially in population over the age of 65 [1]. As a chronic musculoskeletal disease, OA is typified by the degenerative loss of articular cartilage and the occurrence of inflammation. Clinically, patients with OA often suffer from pain, loss of mobility and a shortened working life [2]. Unfortunately, the current drugs for OA are partially effective, leading to limited therapeutic strategy for OA patients with only relief of pain or eventual joint replacement [3].

Cartilage homeostasis is critical for the functionality of joints, and it will tilt toward disruption during the progression of OA [4]. Chondrocyte has been validated as a pivotal participator in maintaining cartilage integrity by regulating the balance of cartilage matrix synthesis and degradation. IL-1β is a critical inflammatory cytokine that has been implicated in the pathogenesis of OA. Once stimulation with IL-1β, chondrocytes will evoked the production of catabolic enzyme matrix metalloproteinases (MMPs) and restrain the synthesis of type II collagen, resulting in cartilage degradation [5,6]. In the text of OA, exposure to IL-1β also induces the release of inflammatory cytokines and mediators, such as prostaglandin E2 (PGE2) and NO, ultimately aggravating inflammatory response in OA [7,8]. Emerging evidence supports a potential therapeutic approach for OA by reducing IL-1β-induced cartilage matrix destruction and inflammatory response [8–10]. Therefore, IL-1β is, theoretically, an excellent candidate for drug targeting OA. However, the clinical trials have shown the unsatisfactory efficacy of IL-1β antagonist and antibody for the treatment of OA [11].

Long non-coding RNAs (lncRNAs) account for more than 90% of human genome and constitute a new class of non-coding RNAs with a length of over 200 nucleotides (nt). LncRNAs can regulate gene expression at various levels, including transcriptional repression, stability mediation and alternative splicing [12]. A growing body of evidence has identified lncRNA as a natural sponge of miRNAs and ultimately harbors extensive miRNA targets. In addition to involve in regulation of normal development, lncRNAs is also associated with the pathogenesis of various diseases such as cancers [13]. The lncRNA plasmacytoma variant translocation 1 (PVT1) is located at 8q24.21 and has been validated to be dysregulated in a variety of diseases. For instance, cessation of PVT1 inhibited osteosarcoma cell proliferation and migration by targeting miR-195, implying a therapeutic target against osteosarcoma [13]. Furthermore, treatment with PVT1 enhanced LPS-induced inflammatory response and ultimately aggravated septic acute kidney injury [14]. Intriguingly, activation of PVT1 by autophagy could protect against hippocampal neuron injury and ultimately ameliorate cognitive impairment in diabetic mice [15]. Recently, microarray analysis confirmed the up-regulation of PVT1 in patients with OA [16]. However, its role in the progression of OA remains poorly elucidated.

In the present study, we detected the expression of PVT1 in OA tissues and IL-1β-stimulated osteoarthritis chondrocytes. Furthermore, the effects of PVT1 on chondrocyte metabolic dysfunction of extracellular matrix and inflammatory response were investigated. The underlying molecular mechanism was also explored.

## Materials and methods

### Cartilage specimen collection and ethics statement

Articular cartilage was aseptically prepared from OA patients undergoing total endoprothesis surgery (*n*=25, including 15 men and 10 women, aged 30–60 years). The normal cartilage specimens were obtained from 25 donors (14 men and 11 women, aged 32–59 years) following trauma or death. No significant differences in age and sex existed among the control and OA groups. Informed consents had been gained from all patients or families before specimen collection. All samples of cartilage were collected from knees and stored at −80°C after promptly frozen by liquid nitrogen. All protocols were performed according to the Declaration of Helsinki, and the experiments were approved by the Ethics Committee of the Ninth Hospital of Xi’an.

### Isolation and culture of primary chondrocytes

To prepare the primary chondrocytes, the collected cartilage was minced and pre-treated with trypsin for 10 min. Then, the tissue slices were digested overnight with collagenase II in DMEM medium containing 10% fetal calf serum (FCS). The isolated cells were then passed through a filter to remove the residual cartilage matrix fragments, followed by centrifugation at 2000 ***g*** for 5 min. Afterward, cells were resuspended in DMEM medium supplemented with 10% FCS and antibiotics consisted of 100 U/ml penicillin and 100 µg/ml streptomycin. Chondrocytes between passages 1 and 3 were included in subsequent experiments. All cells were housed in a humidified atmosphere with 5% CO_2_ at 37°C.

### Constructs, oligonucleotide synthesis and transfection assay

Human PVT1 sequence was PCR amplified and subcloned into the *Xho* I and *BamH* I cloning site of the pcDNA3.1(+) vector (Invitrogen, Carlsbad, CA) to construct the recombinant pcDNA-PVT1 plasmids. The siRNA fragments targeting PVT1 and negative control (NC) were referenced [13] and synthesized by GenePharma (Shanghai, China). Oligonucleotide sequences of miR-149 inhibitor (anti-miR-149), inhibitor control (anti-NC), miR-149 mimics and negative control (NC) were also obtained from GenePharma. Cells were transfected with the recombinant plasmids or oligonucleotides using the Lipofectamine 2000 reagent (Invitrogen, Carlsbad, California, U.S.A.) according to the protocol provided by the manufacturers.

### Real-time quantitative RT-PCR (qRT-PCR)

Total RNA was extracted from OA tissues and chondrocytes exposed to IL-1β (10 ng/ml) for 24 h using the TRIzol reagent (Invitrogen). Then, reverse transcription was performed to synthesize the first cDNA using the SuperScript II First Strand Synthesis System (Invitrogen). Afterward, the obtained cDNA (2 µl) was subjected to real-time PCR to evaluate the transcript levels, including PVT1, miR-149, aggrecan, collagen II, MMP-3, MMP-9 and MMP-13. All procedures were performed according to the instruction of the SYBR Premix Ex Taq™ II Kit (Takara Bio Inc., Otsu, Japan). The specific primers for them were obtained from Sangon Biotech (Shanghai) Co., Ltd. (Shanghai, China) as previously reported [5,17,18]. All reactions were carried out on the ABI PRISM 7000 sequence detection system (Applied Biosystems, Foster City, CA). For normalization, U6 was used as an internal reference for miR-149, and β-actin was introduced for others. The relative transcript levels were calculated according to the 2^−ΔΔ*C*^_t_ method.

### Western blotting analysis

Cells upon various treatments were homogenized and lysed with RIPA lysis buffer. The extracted protein concentration was detected by the BCA protein kit (Beyotime, Shanghai, China). Then, equal amounts of protein were loaded onto 12% SDS-PAGE gel, and then were transferred into a PVDF membrane. After incubation with 5% non-fat milk for 1.5 h to interdict the non-specific binding, the membrane was then immunoblotted overnight at 4°C with the primary antibodies against human aggrecan and collagen II (both from Abcam, Cambridge, MA, U.S.A.). Horseradish peroxidase-conjugated second antibody was then added for further incubation. One hour later, the binding signals were visualized by ECL reagent (Beyotime). The densitomeric assay of bands was performed using a Gel Doc^™^ XR imaging system (Bio-Rad Laboratories, Hercules, CA, U.S.A.) and Quantity One software. β-Actin was used as a normalization to control.

### Determination of NO production

After treatment with various conditions, cells were performed with Griess reaction to detect NO content using the NO detection kit (Nanjing Jiancheng Bioengineering Institute, Nanjing, China). Approximately 10 min later, nitrite was spectrophotometrically captured at 550 nm with reference to a freshly prepared nitrite standard curve.

### ELISA assay

Cells were transfected with various plasmids, siRNA or miR-149, and then were exposed to IL-1β (10 ng/ml) for 24 h. After that, the levels of PGE2, MMP-3, MMP-9, MMP-13, IL-6, IL-8 and TNF-α in culture medium were measured using the commercial ELISA kits (ebioscience, San Diego, CA, U.S.A.) according to the manufacturer’s standard protocols.

### Luciferase reporter assay

For luciferase reporter experiments, the PVT1 wild-type (PVT1-wt) and the mutant derivative lacking of the miR-149-binding site (PVT1-mut) were synthesized and inserted into pGL3 vector (Promega, Madison, WI, U.S.A.) downstream of the luciferase coding region. Human genomic sequences (200 bp) were reversely inserted into the pGL3 vector to construct the miR-149 sensor reporter as previously described [19]. Then, cells were co-transfected with constructs and pRL-TK (Promega), and miR-149 mimics or miR-NC using Lipofectamine 2000. Forty-eight hours later, luciferase activity signals were analyzed using the dual-luciferase reporter assay system (Promega).

### Pull-down assay with biotinylated PVT1 probe

The biotinylated DNA probe complementary to PVT1 RNA was synthesized by GenePharma. Then, the probes were dissolved in binding/washing buffer including 20 mM Tris-HCl (pH 7.5), 0.5 M NaCl and 1 mM EDTA. To generate probe-coated magnetic beads, the probes were cultured with streptavidin-coated Dynabeads M-280 (Invitrogen) for 15 min. After that, chondrocyte lysates were treated with above probe-coated beads. The binding RNA complexes were eluted and exacted for Northern blot assay. The PVT1 pull-down probe sequence was 5′-Bio-ATCCTTTCCGCAAGGAAATC-3′; and random pull-down probe sequence used as negative control was 5′-Bio-CTCGGAGCCAGAATTCTTTC-3′.

### Northern blot assay

After running on a 15% polyacrylamide–urea gel, all samples were transferred to positively charged nylon membranes (Millipore, Bedford, MA, U.S.A.). The samples were cross-linked by UV irradiation, and then were hybridized with 100 pmol 3′-digoxigenin (DIG)-labeled probes for miR-149 (5′-CAAGCACGGGAGTGAAGACA-3′) overnight at 42°C. The revelation of the hybridization was then evaluated using a DIG luminescent detection kit (Roche, Mannheim Germany) as per the manufacturer’s protocols. U6 was introduced as an internal standard, and its probe sequence was 5′-TGTGCTGCCGAAGCGAGCAC-3′.

### Pull-down analysis with biotinylated miR-149

Chondrocytes were transfected with 50 nM biotinylated miR-149 or the mut (GenePharma) for 48 h. After rinsing with cold PBS, cells were lysed with lysis buffer (Promega). Afterward, one half of specimen was aliquoted for input. The other samples were incubated with M-280 streptavidin-coated magnetic beads (Invitrogen) at 4°C. Three hours later, the samples were washed twice with ice-cold lysis buffer and subsequent rinsed three times with low-salt buffer. Then, the specimens were rinsed with high-salt buffer containing 0.1% SDS, 2 mM 193 EDTA, 1% Trition X-100, 500 mM NaCl, 20 mM Tris-HCl with pH 8.0. The bound RNAs were then extracted using TRIzol reagent and subjected into qRT-PCR assay to detect lncRNA PVT1 enrichment.

### Statistical analysis

All data from at least three independent experiments were presented as the mean ± standard deviation (SD). Results were analyzed by the SPSS 19.0 software (SPSS Inc., U.S.A.). Statistical analysis was carried out using *t*-test for two groups and ANOVA for three or more groups, followed by the Tukey’s *post-hoc* test. *P*<0.05 was considered to indicate statistical significance.

## Results

### Expression of PVT1 is elevated in human OA articular cartilage and IL-1β-stimulated chondrocytes

**Figure 1:**
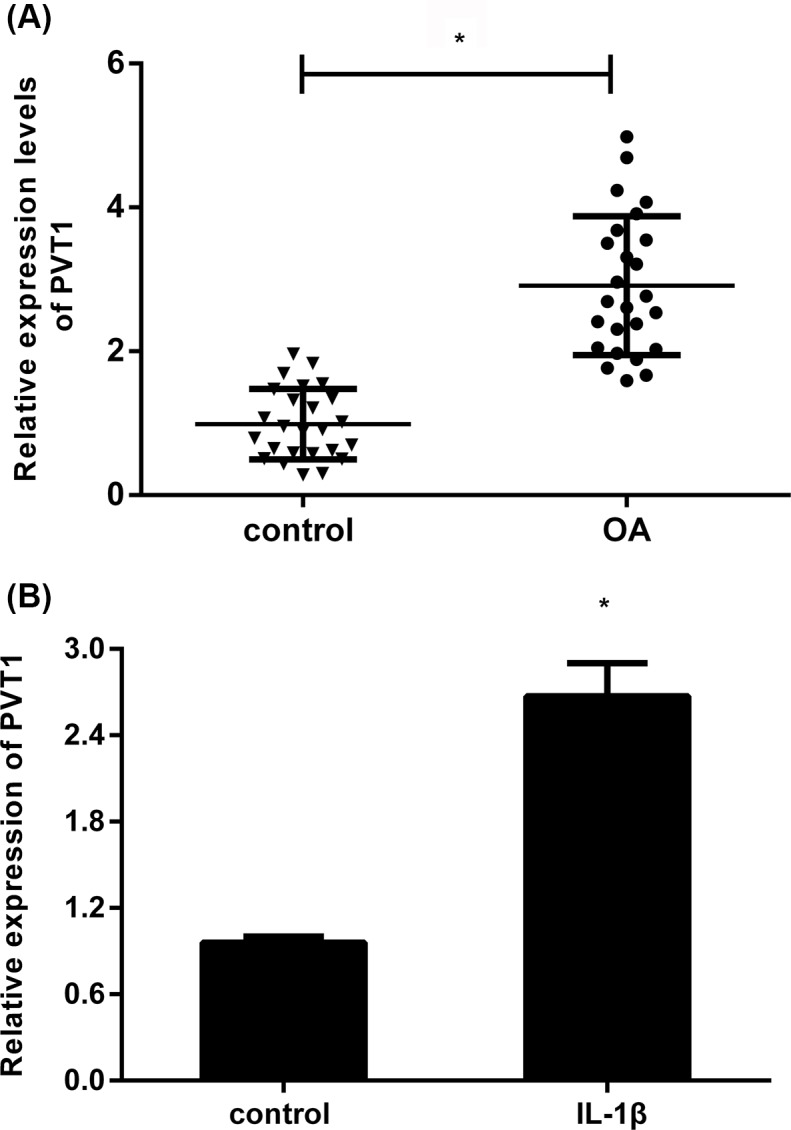
Expression of PVT1 increased in human OA articular cartilage and IL-1β-stimulated chondrocytes (**A**) The expression levels of PVT1 in human OA tissues were determined by qRT-PCR. (**B**) After exposure to IL-1β (10 ng/ml) for 24 h, the expression of PVT1 in OA chondrocytes was also detected. *P<0.05 vs. control group.

To explore the role of PVT1 in the pathological progression of OA, we first detected its expression in 25 pairs of OA and normal tissues. QRT-PCR assay confirmed that the RNA levels of PVT1 were higher in OA tissues relative to control groups (Figure 1A). Moreover, stimulation with inflammatory cytokine IL-1β, a critical culprit in the development of OA, dramatically elevated the expression of PVT1 in contrast with control groups (Figure 1B). These results indicate a potential predominant role of PVT1 in OA.

### Cessation of PVT1 ameliorates metabolic dysfunction in chondrocytes upon IL-1β condition

**Figure 2:**
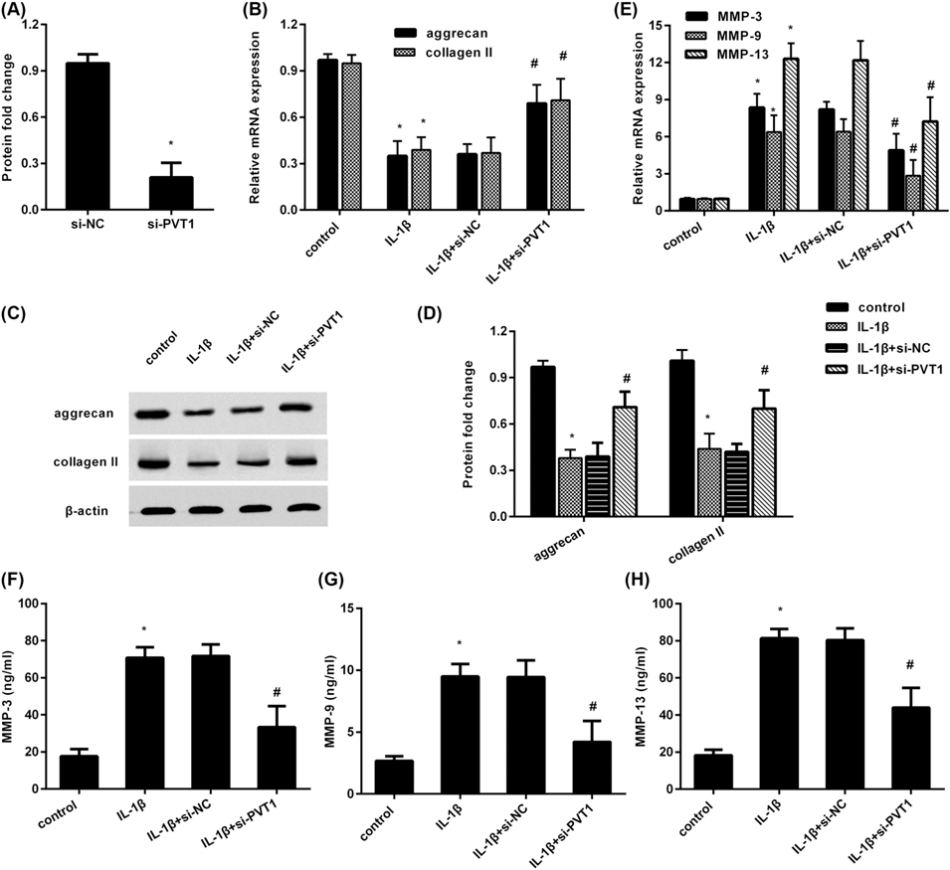
PVT1 inhibition antagonized the metabolic imbalance toward catabolism in chondrocytes upon IL-1β stimulation (**A**) Cells were transfected with si-PVT1 or si-NC for 48 h. The expression of PVT1 was determined. *P<0.05 vs. si-NC-treated group. (**B**) After transfection with si-PVT1, cells were exposed to IL-1β. Then, the mRNA levels of aggrecan and collagen II were measured by qRT-PCR. (**C**) The corresponding protein levels of aggrecan and collagen II were analyzed by Western blotting. (**D**) Quantified analysis of protein was performed by Quantity One software. (**E**) The subsequent transcript levels of MMP-3, MMP-9 and MMP-13 were determined by qRT-PCR. (**F**–**H**) The contents of MMP-3 (F), MMP-9 (G) and MMP-13 (H) in supernatants were evaluated by ELISA assay. *P<0.05 vs. control group. ^#^*P*<0.05 vs. IL-1β-treated group.

Abundant research has confirmed metabolic dysfunction of chondrocytes in OA inflammatory environments [5,20]. To elucidate the function of PVT1 in chondrocyte metabolism under inflammatory conditions, the expression of PVT1 was muted in chondrocytes after transfection with PVT1 siRNA (Figure 2A). Importantly, blocking PVT1 expression antagonized IL-1β-induced transcripts of aggrecan and collagen II (Figure 2B), both critical cartilage extracellular matrix components. Simultaneously, the analogous changes in the protein expression of aggrecan and collagen II were also detected after PVT1 silencing (Figure 2C,D). Additionally, exposure to IL-1β increased the mRNA levels of matrix catabolic MMPs, including MMP-3, MMP-9 and MMP-13 (Figure 2E). In striking contrast, these increases were reversely inhibited following PVT1 suppression. Furthermore, the contents of MMP-3 in supernatants from IL-1β-stimulated chondrocytes were reduced when cells were transfected with PVT1 siRNA (Figure 2F). Concomitantly, PVT1 suppression also attenuated the elevation of MMP-9 (Figure 2G) and MMP-13 (Figure 2H) levels in supernatants from cells upon IL-1β exposure. These data suggest that PVT1 inhibition counteracted IL-1β-triggered metabolic dysfunction toward catabolism, implying a pivotal role of PVT1 in cartilage degradation.

### PVT1 suppression antagonizes IL-1β-triggered inflammatory response

**Figure 3:**
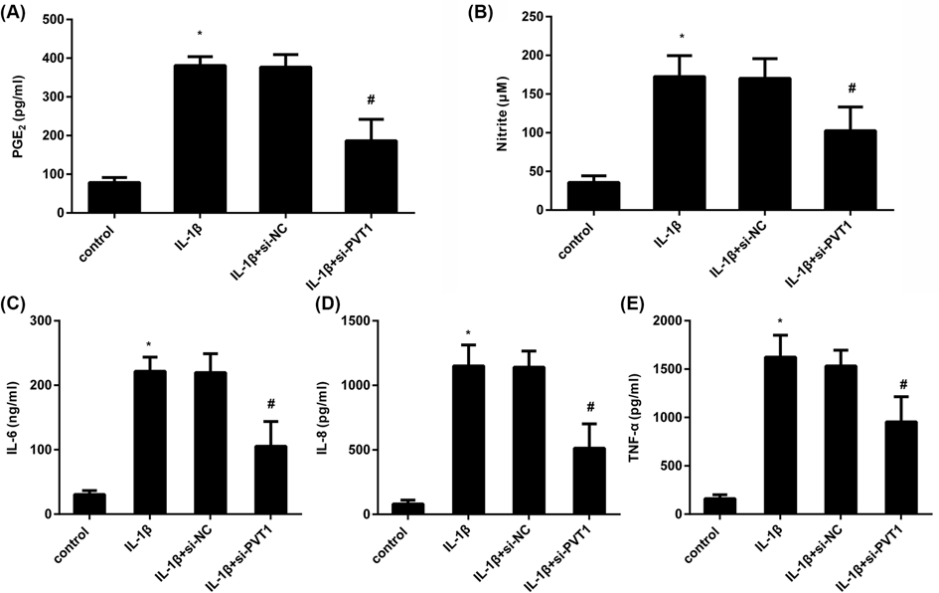
Cessation of PVT1 inhibited IL-1β-induced inflammatory response (**A**) Chondrocytes were transfected with si-PVT1, prior to exposure to IL-1β for 24 h. Then, the concentration of PGE2 in supernatants was detected by ELISA. (**B**) The production of NO was analyzed by a NO detection kit. (**C**–**E**) The inflammatory cytokine levels of IL-6 (C), IL-8 (D) and TNF-α (E) in culture medium were also measured by ELISA. *P<0.05 vs. control group. ^#^*P*<0.05 vs. IL-1β-treated group.

Next, we investigated the effects of PVT1 on IL-1β-induced inflammatory response. As shown in Figure 3A, IL-1β stimulation enhanced the production of PGE2, which was abrogated by PVT1 inhibition. Moreover, blocking PVT1 expression also reversed the elevation of NO concentration in supernatants of IL-1β-treated cells (Figure 3B). Additionally, in contrast with the control groups, IL-1β exposure enhanced the release of pro-inflammatory cytokines, including IL-6 (Figure 3C), IL-8 (Figure 3D) and TNF-α (Figure 3E). However, these increases in inflammatory cytokines were counteracted after PVT1 inhibition.

### PVT1 regulates miR-149 expression and activity

**Figure 4:**
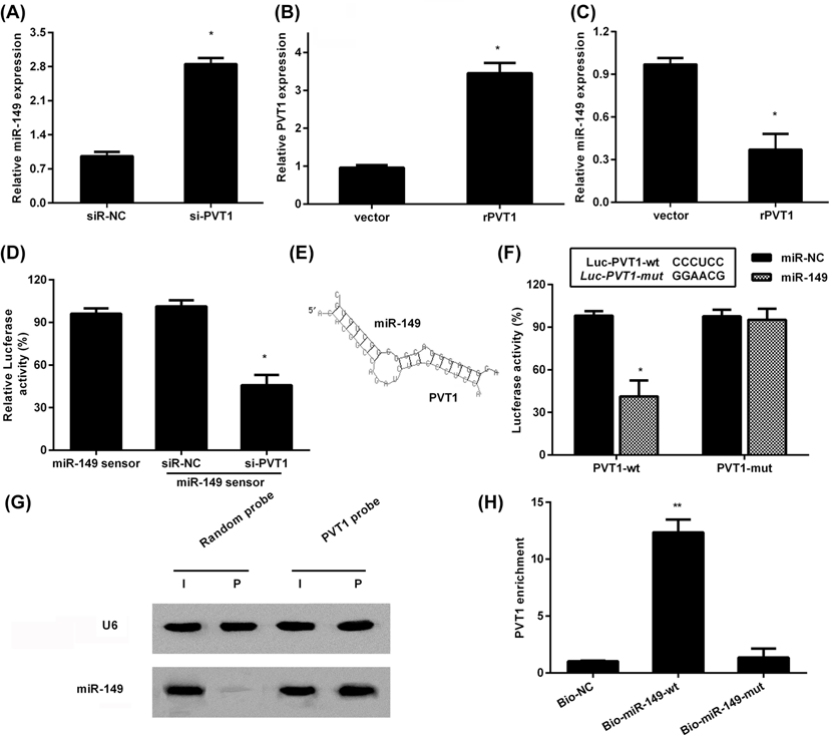
PVT1 directly bound to miR-149 and regulated its expression (**A**) Following transfection with si-PVT1, the expression of miR-149 was assessed by qRT-PCR. *P<0.05 vs. si-NC-treated group. (**B**) Cells were transfected with recombinant PVT1 (rPVT1) plasmids, then the expression of PVT1 was detected. (**C**) The expression of miR-149 in PVT1-overexpressed cells. *P<0.05 vs. vector-treated group. (**D**) Cells were treated with si-PVT1 or si-NC, and then were transfected with miR-149 sensor. After that, the luciferase activity was measured. (**E**) Schematic illustration of the presumed site complementary to miR-149. (**F**) After co-transection with Luc- PVT1-wt, or Lcu-PVT1-mut, and miR-149 mimics. Luciferase activity was then assessed. (**G**) miR-149 was pulled down using biotinylated PVT1 probe or random probe. Then, the expression levels of miR-149 were detected by Northern blot. (**H**) PVT1 is associated with miR-149. Cells were transfected with biotinylated miR-149 wt (Bio-149-wt) or its mutant (Bio-149-mut). The negative control (Bio-NC) means that a biotinylated microRNA that is not complementary to PVT1. Approximately 48 h later, the expression of PVT1 was evaluated by qRT-PCR; *P<0.05, **P<0.01.

LncRNAs have been widely accepted as a critical participator in inflammation-related diseases including OA by acting as the competing sponge RNAs to bind and sequester miRNAs [13,21]. miR-149 exerts an important role in regulation of inflammation and MMP production and was recently validated to be down-regulated in patients with OA [22,23]. To elucidate the mechanism underlying the role of PVT1 in IL-1β-mediated catabolism and inflammatory response, we detected the expression of miR-149. As shown in Figure 4A, cessation of PVT1 notably increased the expression of miR-149 in IL-1β-treated cells. Moreover, transfection with recombinant PVT1 plasmids enhanced the expression of PVT1 (Figure 4B). Conversely, overexpression of PVT1 reduced miR-149 levels (Figure 4C). To further explore the effect of PVT1 on the activity of miR-149, we constructed a miR-149 sensor, and depression of PVT1 dampened luciferase activity of sensor, indicating that PVT1 silencing induces miR-149 activity (Figure 4D).

### PVT1 directly interacts with miR-149

To explore the precise mechanism by which PVT1 modulates miR-149 levels, we then investigated whether PVT1 could interact with miR-149. After comparing the sequence of PVT1 with that of miR-149 by bioinformatics program RNAhybrid, we demonstrated that PVT1 indeed contained a potential binding site for miR-149 (Figure 4E). Moreover, miR-149 up-regulation muted the luciferase activity of PVT1 in chondrocytes, but with little change in PVT1 mutation-treated groups (Figure 4F). Additionally, biotin-labeled pull-down assay confirmed that a biotin-labeled specific PVT1 probe could pull-down miR-149 (Figure 4G). Consistently, after transfection with biotinylated miR-149, the subsequent biotin-based pull-down analysis found that PVT1 was pulled down by miR-149 via qRT-PCR assay, which did not occur in miR-149-mut groups (Figure 4H). All results reveal that PVT1 can directly bind to miR-149.

### Depression of miR-149 alleviates PVT1 inhibition-protected against aberrant metabolic dysfunction and inflammation in IL-1β-simulated chondrocytes

**Figure 5:**
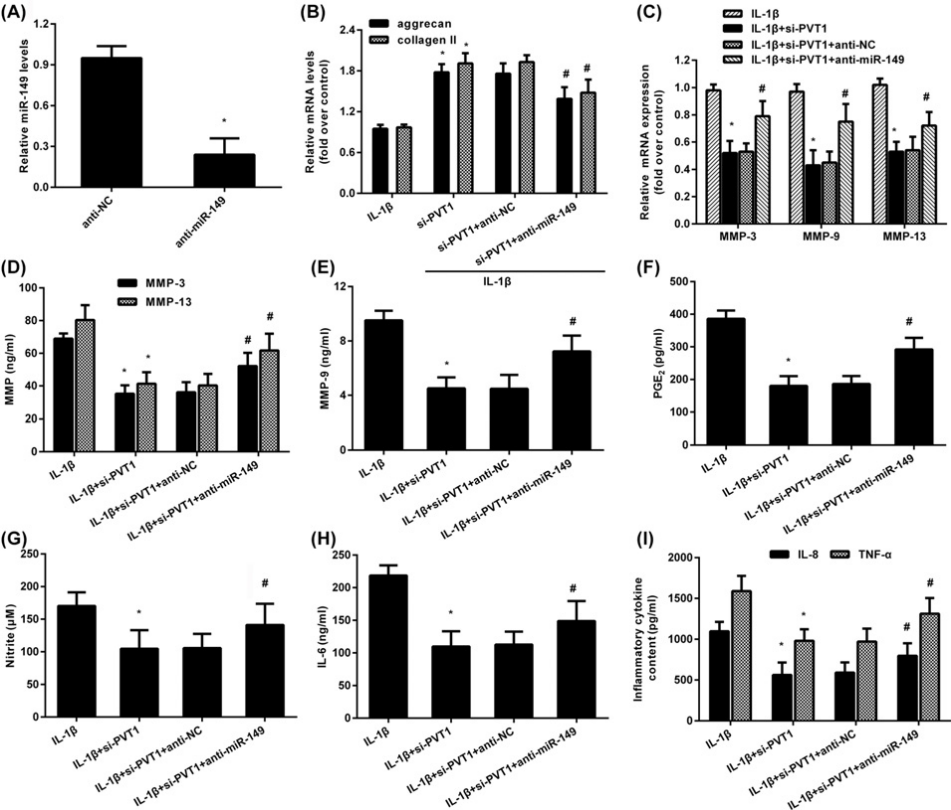
miR-149 suppression reversed PVT1 cessation-inhibited aberrant catabolism and inflammation triggered by IL-1β stimulation (**A**) The transfected efficiency of miR-149 inhibitor. (**B**) Cells were transfected with si-PVT1 and anti-miR-149 inhibitor, following the exposure to IL-1β. Then, the mRNA levels of aggrecan and collagen II were determined by qRT-PCR. (**C**) The corresponding effects on the transcripts of MMPs. (**D**–**I**) The subsequent levels of MMP-3 and MMP-13 (D), MMP-9 (E), PGE2 (F), NO (G), IL-6 (H), IL-8 and TNF-α (I) in culture supernatants were also detected. *P<0.05 vs. control group. ^#^*P*<0.05 vs. si-PVT1 group.

**Figure 6:**
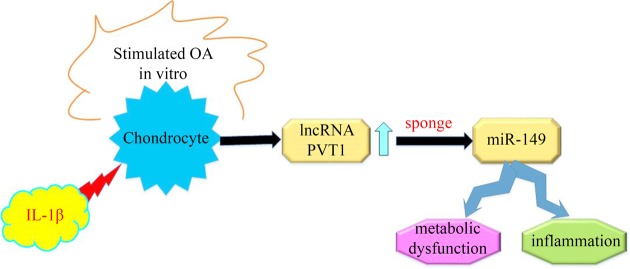
The graphical representation of this manuscript

We further elucidated the role of miR-149 in PVT1 cessation-mediated protective role in attenuating IL-1β-evoked excessive catabolism and inflammatory response. Chondrocytes transfected with miR-149 inhibitors presented an obvious decrease in miR-149 expression (Figure 5A). Moreover, PVT1 suppression-elevated transcripts in aggrecan and collagen II were mitigated after miR-149 down-regulation in IL-1β-treated cells (Figure 5B). Furthermore, miR-149 suppression also antagonized the inhibitory effects of PVT1 cessation on MMP-3, MMP-9 and MMP-13 levels (Figure 5C), concomitant with similar increases in their concentration in supernatants (Figure 5D,E). Simultaneously, abrogating miR-149 expression also reversed the adverse roles of PVT1 inhibition in PGE2 (Figure 5F) and NO (Figure 5G) levels under IL-1β condition. Moreover, PVT1 suppression-restrained productions of inflammatory cytokines IL-6 (Figure 5H), IL-8 and TNF-α (Figure 5I) were also counteracted following miR-149 decline in IL-1β-treated cells. These data suggest that PVT may regulate aberrant metabolic dysfunction and inflammation in IL-1β-simulated chondrocytes by sponging to miR-149 (Figure 6).

## Discussion

During the past few years, lncRNAs have received increasing interest as their involvement in various pathogenic processes, including cancer, cardiovascular and inflammatory diseases. Recent research has supported the critical role of lncRNAs in joint-related diseases that may open up a promising opportunity for developing novel therapeutic targets [16,21]. Specially, PVT1 affords the predominant intervention in response to various cellular processes, such as cell injury, invasion and inflammatory response [14,15,24]. For instance, previous research found that PVT1-mediated autophagy could protect against hippocampal neuron apoptosis and thereby alleviate cognitive impairment in diabetic mice [15]. In the present study, we observed the obvious elevation of PVT1 in OA patients, which is consistent with previous report [16]. Furthermore, IL-1β, a critical contributor to the development of OA, also increased the expression of PVT1. Therefore, these results indicate a potential crucial role of PVT1 in the progression of OA.

The hallmark feature during OA is the destruction of articular cartilage, resulting from the dysfunction of cartilage homeostasis toward catabolism [1,18]. It is known that extracellular matrix is constituted primarily by collagen, glycoproteins and proteoglycan, and will be degraded by the production of matrix-degrading enzyme MMPs. In normal context, extracellular matrix generally maintains a dynamic balance between anabolism and catabolism in articular cartilage. However, this balance will be lost under pathologic conditions and incurs the joint-related diseases including OA. Therefore, to explore the role of PVT1 in OA, we investigated its function in chondrocyte metabolic dysfunction. Similar to previous study [6,20], exposure to IL-1β inhibited the expression of matrix synthesis-related molecules and increased MMP production, which may aggravate matrix degradation. Intriguingly, cessation of PVT1 reversed the inhibitory effect of IL-1β on aggrecan and collagen II expression in chondrocytes. Simultaneously, the production of MMP-3, MMP-9 and MMP-13 upon IL-1β stimulation was also counteracted after PVT1 suppression. Accordingly, these results suggest that PVT1 inhibition may ameliorate the pathologic progression of OA by attenuating chondrocyte aberrant metabolism toward catabolism via suppressing matrix deposition and promoting catabolic enzyme production. Loss of chondrocytes usually results in the insufficient cartilage cell number and ultimately contributes to functional loss in cartilage. Previous finding confirmed that inhibition of PVT1 restrained the apoptosis of osteoarthritic chondrocytes [25].

Inflammation is widely accepted as a critical driver of OA and affects the pathogenic progression and pain by releasing various inflammatory cytokines and mediators [7,9,26]. Among these cytokines, IL-1β exerts a pivotal role in the development of OA. Increased levels of IL-1β have been validated in the synovial fluid and cartilage tissues of patients with OA [27]. Stimulation with IL-1β in OA chondrocytes evokes the production of MMPs and inflammatory cytokines that further aggravate inflammatory response and contribute to the pathogenesis of OA [9,26]. An increasing body of evidence has supported a promising therapeutic strategy against OA by attenuating the inflammatory response [8,10]. Here, the induction of inflammatory mediators by IL-1β was confirmed including IL-6, IL-8, TNF-α, PGE2 and NO, which has been extensively reported in previous research [7,20]. More importantly, our finding confirmed that blocking PVT1 expression antagonized IL-1β-triggered inflammatory response. Intriguingly, overexpression of PVT1 promoted inflammatory response in LPS-induced septic acute kidney cells by elevating the releases of TNF-α, IL-6 and IL-1β [14].

MicroRNAs (miRNAs) are highly conserved small non-coding RNA molecules and represent a new class of post-transcriptional regulators that can silence target genes by interacting with the 3’-untranslated region. Mounting evidence substantiates the abnormal expression of miRNAs during the development of degenerative joint diseases including OA [23,28]. LncRNAs has been proved to act as miRNAs ‘sponges’ to regulate miRNA expression and activity. Abundant research confirms that lncRNAs are involved in a variety of pathogenic processes by interacting with miRNA. Specially, many studies have confirmed the correlation between various miRNAs and OA, such as miR-149. Previous evidence corroborated the down-regulation of miR-149 in OA cartilage and osteoarthritic chondrocytes [22,23]. In this study, we found that PVT1 overexpression suppressed miR-149 expression, which was inversely increased after PVT1 cessation. More intriguingly, PVT1 could directly bind to miR-149 and inhibit its activity. It has been validated that exposure to IL-1β reduces the expression of miR-149, which accounts for the elevation pro-inflammatory cytokines such as TNF-α, IL-1β and IL-6 [23]. Previous study found that miR-149 could act as an inflammatory inhibitor to repair the scarless wound [29]. Additionally, elevation of miR-149 restrained the expression of MMP-9 and MMP-2 in cancer cells [17,30]. We further explored whether miR-149 is involved in PVT1 silencing-mediated protective roles against IL-1β-induced metabolic malfunction and inflammation. As expected, blocking miR-149 expression reversed the inhibitory effects of PVT1 suppression on matrix degradation and inflammatory cytokine levels.

## Conclusions

In summary, the present study corroborated the elevation of PVT1 in OA cartilage and IL-1β-stimulated OA chondrocytes. More importantly, PVT1 silencing antagonized metabolic imbalance toward catabolism and inflammation in response to IL-1β exposure by acting as a sponge of miR-149. Therefore, this research elucidates how PVT1 aggravates the development of OA, supporting a promising therapeutic agent against degenerative joint diseases including OA.

## Abbreviations

DIG: 3′-digoxigenin
DMEM: Dulbecco’s modified Eagle’s medium
IL: interleukin
lncRNA: long non-coding RNA
MMP: matrix metalloproteinase
NO: nitric oxide
OA: osteoarthritis
PGE2: prostaglandin E2
PVT1: plasmacytoma variant translocation 1
TNF-α: tumor necrosis factor α
